# Subretinal Fluid in Eyes with Active Ocular Toxoplasmosis Observed Using Spectral Domain Optical Coherence Tomography

**DOI:** 10.1371/journal.pone.0127683

**Published:** 2015-05-26

**Authors:** Yanling Ouyang, Fuqiang Li, Qing Shao, Florian M. Heussen, Pearse A. Keane, Nicole Stübiger, Srinivas R. Sadda, Uwe Pleyer

**Affiliations:** 1 Charité, University Medicine Berlin, Department of Ophthalmology, Berlin, Germany; 2 Eye Center affiliated with 2nd Hospital, Jilin University, Changchun, China; 3 NIHR Biomedical Research Centre for Ophthalmology, Moorfields Eye Hospital NHS Foundation Trust and UCL Institute of Ophthalmology, London, United Kingdom; 4 Doheny Eye Institute and Department of Ophthalmology, Los Angeles, California, United States of America; Duke University, UNITED STATES

## Abstract

**Purpose:**

To describe the clinical finding of subretinal fluid (SRF) in the posterior pole by spectral domain optical coherence tomography (SD-OCT) in eyes with active ocular toxoplasmosis (OT).

**Design:**

Retrospective case series.

**Participants:**

Thirty-eight eyes from 39 patients with active OT.

**Methods:**

Eyes with active OT which underwent SD-OCT were reviewed. SRFs in the posterior pole were further analyzed.

**Main Outcome Measures:**

Presence of SRF; its accompanying features, e.g. retinal necrosis, cystoid macular edema (CME), choroidal neovascularization (CNV); and longitudinal changes of SRF, including maximum height and total volume before and after treatment.

**Results:**

SRF presented in 45.5% (or 15/33) of eyes with typical active OT and in 51.3% (or 20/39) of eyes with active OT. The mean maximum height and total volume of SRF were 161.0 (range: 23–478) µm and 0.47 (range: 0.005–4.12) mm3, respectively. For 12 eyes with SRF related to active retinal necrosis, SRF was observed with complete absorption after conventional anti-toxoplasmosis treatment. The mean duration for observation of SRF clearance was 33.8 (range: 7–84) days. The mean rate of SRF clearance was 0.0128 (range: 0.0002–0.0665) mm3/day.

**Conclusions:**

SRF (i.e., serous retinal detachment) is a common feature in patients with active OT when SD-OCT is performed. The majority of SRF was associated with retinal necrosis and reacted well to conventional therapy, regardless of total fluid volume. However, SRF accompanying with CME or CNV responded less favorably or remained refractory to conventional or combined intravitreal treatment, even when the SRF was small in size.

## Introduction

Ocular toxoplasmosis (OT) is the most common etiology of infectious posterior uveitis in otherwise healthy individuals, leading to legal blindness in at least one eye in approximately 25% of patients [1,2]_._ It is caused by Toxoplasma gondii and frequently affects children and young adults, with significant morbidity, and thus has considerable socio-economic implications. The disease characteristics vary in different areas of the world [3]. Active OT consists of well-defined foci of coagulative necrosis of the retina. Toxoplasma gondii antigens are often detected in areas of necrosis by immunohisto- chemistry. Diagnosis of OT is based on clinical characteristics consistent with toxoplasmic retinochoroiditis (foci of retinal necrosis with associated retinal inflammation), in the absence of other identifiable causes [3]. In recent years, clinically tailored laboratory analysis, including serology and/or aqueous humor analysis (detection of intraocular Toxoplasma gondii antibodies with Goldmann/Witmer coefficient >3) was also used to aid the diagnosis of OT for eyes with atypical clinical manifestations [2].

Subretinal fluid (SRF) or exudative/serous retinal detachment (RD) is commonly observed in posterior uveitis. The effect of SRF on vision and its response to therapy in uveitis patients has been an active field of interests in recent years [4]_._ However, the frequency and visual impact of SRF in OT have not been well defined. The early observation of SRF in patients with OT can be traced back to 1960s, when the parasite was successfully isolated from SRF [5]_._ Since then, SRF in OT has been mainly reported in the literature anecdotally as case reports or briefly mentioned in the case series [6–10]_._

Optical coherence tomography (OCT) provides high-resolution cross-sectional images of the neurosensory retina and has been proven to be useful in the management of uveitis. In patients with serous RD, OCT parameters can be a reliable real-time indicator of the severity of the inflammation and the effectiveness of the treatment. These parameters can be more reliable than the visual acuity in assessing response to intervention [11]_._ Widespread availability of spectral domain OCT (SD-OCT) has provided improved visualization of the details of the neurosensory retina and has facilitated the detection of many retinal/choroidal abnormalities, including cystoid lesions in OT and SRF [10, 12, 13]_._ The purpose of the current study is to report the observation of SRF in patients with active OT, and to investigate its associate clinical course and therapeutic consequences.

## Materials and Methods

### Baseline Data Collection

**Fig 1:**
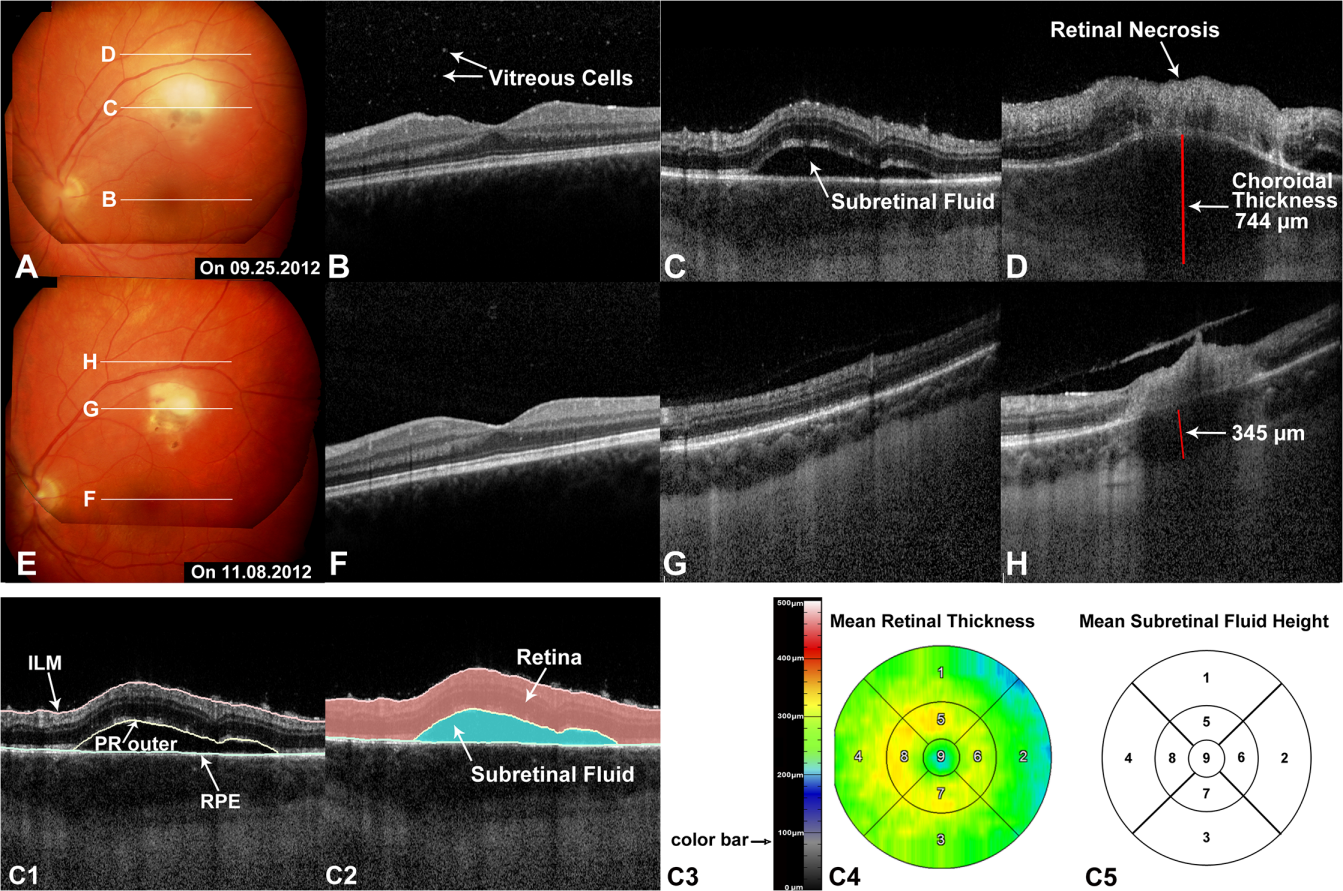
Subretinal fluid (SRF) in typical active ocular toxoplasmosis as seen by optical coherence tomography (OCT) (Patient 9, Table 1). **Parts A-D**: fundus photos (FP) and OCT images on the initial visit. **Part A**: a fresh whitish inflammatory lesion superior to the macula in the extramacular area, with intraretinal hemorrhage and SRF surrounding the necrosis. **Parts B-D**: OCT B-scans corresponding to the lines B-D shown in Part A. Vitreous cells were present with normal retinal structure through the fovea (Part B). In areas surrounding the active lesion, SRF was visable (Part C-D). Full-thickness retina destruction (retinal necrosis) accompanied by largely increased choroidal thickness (744 μm) was present (Part D). **Parts E-H:** FP and OCT images 6 weeks after the initial visit. **Part E**: previous whitish inflammatory lesion was smaller without visible SRF. **Parts F-H**: OCT B-scans corresponding to the lines F-H shown in Part E. Absence of SRF and dramatically lessened vitreous cells in the macula and area around the active lesion were observed (Part F-H). Retina got thinner, but still with full-thickness necrosis at the previous active site (Part H). The choroid thickness decreased to 345 μm (Part H). **Parts C1-C2** showed the segmentation of retinal layers in OCT B-scan corresponding to Figure C. **Part C3-C5**: quantitative measurement results from manual segmentation. **Part C3:** the color bar showing the correlation of thickness and color range. **Parts C4-C5:** mean retinal thickness and mean SRF height shown in early treatment diabetic retinopathy study (ETDRS) grid using foveola as the center of the grid. The correlation of numbers and subfields are as follows: 1 = superior outer macula; 2 = temporal outer macula; 3 = inferior outer macula; 4 = nasal outer macula; 5 = superior inner macula; 6 = temporal inner macula; 7 = inferior inner macula; 8 = nasal inner macula; 9 = central subfield). ILM = internal limiting membrane; PR = photoreceptor layer; RPE = retinal pigment epithelium.

Data were retrospectively collected from consecutive patients attending the Department of Ophthalmology at Charité, University Berlin, between January 2010 and September 2013, with a diagnosis of OT. Only patients with typical or atypical active episodes related to OT were included. Typical active OT was defined based on the clinical picture of a characteristic newly observed focus of retinitis (example shown in Fig 1-A), which usually arises from the border of a retinochoroidal scar, in the absence of other identifiable causes [3] [14] [15], with or without laboratory confirmation. Atypical OT episode was considered in suspected cases with atypical presentation, who deemed to be positive with the clinically tailored laboratory analysis, including serology and/or aqueous humor analysis (detection of intraocular Toxoplasma gondii antibodies with Goldmann/Witmer coefficient >3) [15]_;_ or who presented with recurrent symptoms in eyes with confirmed previous diagnosis of OT. Patients with acquired immunodeficiency syndrome (AIDS), other immune system related diseases, or individuals receiving immunosuppressive agents (other than corticosteroids specifically for OT) were excluded. For inclusion in the study, all patients with active OT were required to have undergone volume OCT scanning with spectral domain OCT (Spectralis, Heidelberg Engineering, Germany).

Information regarding age, sex, ethnicity, history of ophthalmic diseases or surgeries, ophthalmic diagnosis, and lens status were also collected. Best-corrected visual acuity (VA) was measured using Snellen VA charts. Approval for data collection and analysis was obtained from the institutional review board of the University Göttingen ethics committee as part of the multi-center German “Toxonet” project. The research adhered to the tenets set forth in the Declaration of Helsinki. Written consent was given by the patients for their information to be stored in the hospital database and used for research.

### Characteristics of eyes with activity related to OT by Clinical Observation

Eyes with active episodes related to OT were evaluated and categorized into 3 groups: 1) "typical active OT", defined as eyes with clinical characteristics of a newly observed focus of retinitis (example shown in Fig 1-A), with or without previous diagnosis of OT; 2) "new atypical active OT", defined as lesions without typical characteristics of a focus of retinitis, but confirmed by laboratory testing at the current visit; in eyes without previous diagnosis of OT; 3) "recurrent atypical active OT", defined as eyes with previous diagnosis of OT presenting with newly onset symptoms, without typical clinical manifestation or confirmatory laboratory testing at the current visit.

Clinical characteristics of active OT were first collected from dilated fundus examination and/or fundus photos (FP), or fluorescein angiography (FA) if available. Locations of these lesions were documented for both ‘zone' (as described by Holland et al. [16]) and area (as 'macula involving the fovea'; 'macula not involving the fovea'; 'extramacula within the posterior pole'). In addition, the presence or absence of choroidal neovascularization (CNV) was also noted, as determined by clinical examination and FA.

### OCT Scanning Protocols and Analysis

In each case, macular volume OCT scans were obtained (i.e., image sets centered approximately on the fovea) using Spectralis OCT. If the active lesion affected areas within the posterior pole but outside the macula, including (1) nasal to the optic disc, (2) superior to the superotemporal vascular arcade, (3) inferior to the inferotemporal vascular arcade, and (4) temporal to the macula [17], then the corresponding extramacular volume OCT scans were also captured. Raw OCT data were exported from the imaging system and imported into validated custom grading software (3D-OCTOR, Doheny Image Reading Center (DIRC), Los Angeles, CA) [18] for further review.

### OCT Grading Methodology

**Fig 2:**
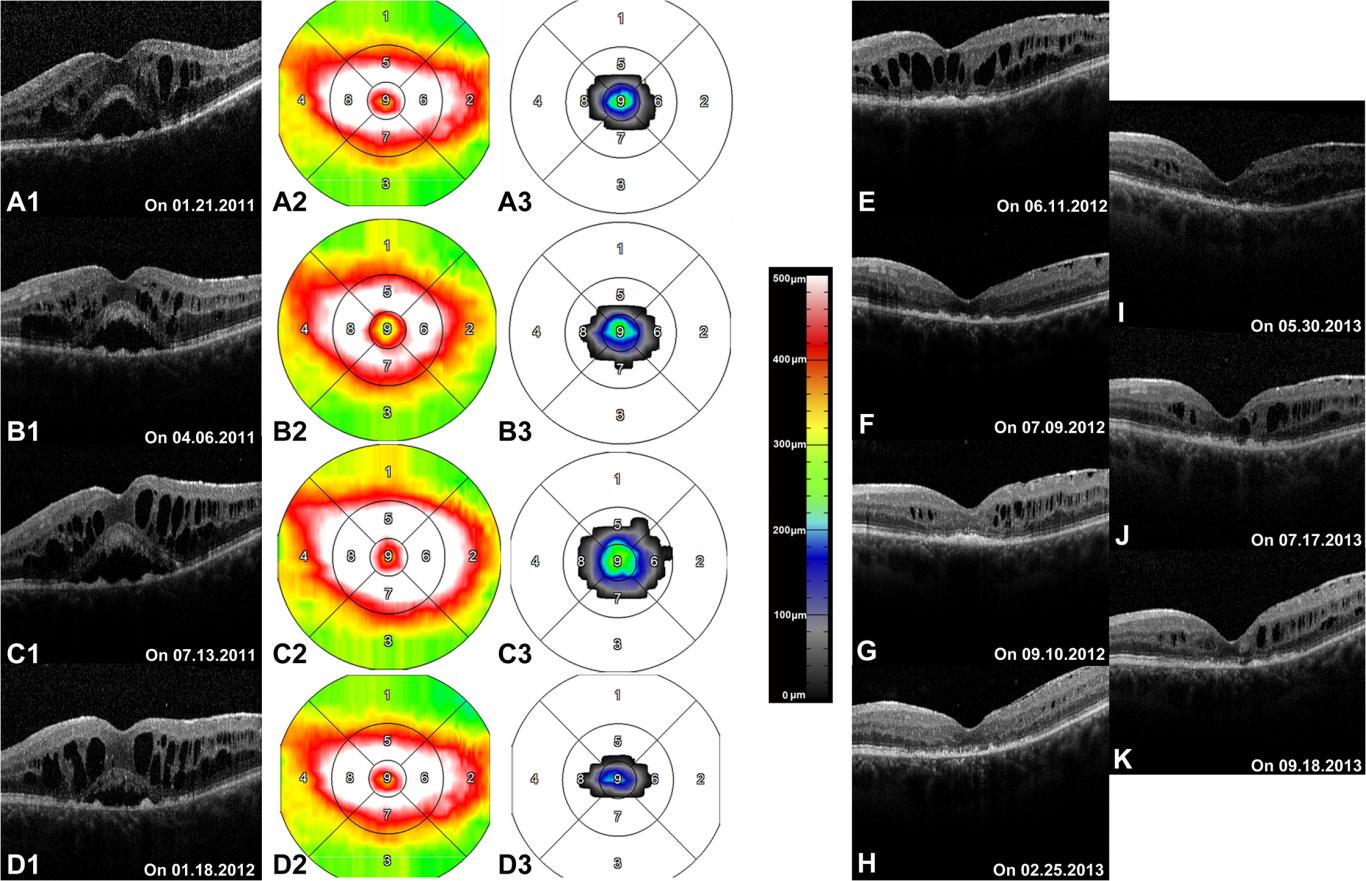
Subretinal fluid (SRF) associated with cystoid macular edema (CME) in one eye with previous diagnosis of ocular toxoplasmosis as seen on optical coherence tomography (OCT) (Patients 16, Table 1). **Parts A1, B1, C1, D1** showed characteristics of SRF and CME seen on OCT B-scans through the fovea. **Parts A2, B2, C2, D2** demonstrated mean retinal thickness maps shown in early treatment diabetic retinopathy study (ETDRS) grid. **Parts A3, B3, C3, D3** were maps of mean SRF height shown in ETDRS grid. SRF and CME kept relatively unchanged for the first 3 visits, and started to respond to treatment on the 4th visit. **Parts E-K** revealed absence of SRF in the following visits, after 3 intraocular injection of 1.25 mg bevacizumab. However, CME remained persistent, although minimization at one time point (H) was temporally seen.

Two graders (Y.O., F.M.H.), certified for the assessment of OCT images at the DIRC, evaluated each OCT image set independently. The presence or absence of SRF was firstly assessed at baseline. When SRF was identified, the co-existence of other features was also documented, including presence or absence of total retinal layer destruction (i.e. retinal necrosis, example in Fig 1-D), cystoid macular edema (CME) (example in Fig 2), and disruption of photoreceptor layer (PR) according to a pre-existing OCT grading methodology [19,20]_._ The location of SRF was also graded as "macula involving the fovea", or "macula not involving the fovea" or "extramacula". For eyes with SRF at baseline, OCT sets in the follow-up visits were also assessed for presence or absence of SRF. Finally, the earliest date of the OCT scans showing SRF clearance was noted.

A reading center open adjudication process was applied to obtain a final consensus result for each case from the dual grading process.

### Quantitative Analysis of OCT Images

For OCT sets containing SRF, manual segmentation was performed, which includes delineation of: internal limiting membrane (ILM) inner boundary, PR outer boundary and retinal pigment epithelium (RPE) inner boundary (example indicated in Fig 1-C1). This segmentation allowed both quantification of retinal thickness (defined as distance between ILM inner and RPE inner boundaries) and multiple measurements of SRF (defined as PR outer to RPE inner boundaries), including maximum and minimum height, area and total volume (example shown in Fig 1-C2). Quantitative parameters were then reported based on the individual subfields or the entire area of the Early Treatment Diabetic Retinopathy Study (ETDRS) grid (example shown in Fig 1-C3, C4 and C5).

### Data Analysis and Statistical Methods

Duration of observed SRF clearance was defined as days between first available OCT date with SRF (as baseline) and the earliest available OCT date showing absence of SRF in the follow-up visits. The rate of SRF clearance was calculated as the total SRF volume at baseline divided by the duration of observed SRF.

Clinical and imaging data were analyzed with frequency and descriptive statistics. Statistical analysis was performed using commercially available software SPSS (version 19.0, SPSS Inc., Chicago, Illinois, United States).

## Results

### Baseline Characteristics

A total of 142 patients with a diagnosis of OT were seen in our tertiary center within the study period, of which, 40 eyes from 39 patients with active episodes related to OT had undergone volume OCT scanning and were reviewed in the study. Among them, one patient was excluded due to an associated diagnosis of AIDS. As a result, 39 eyes from 38 patients were included in the study, comprising 33 eyes with typical active OT, 1 eye with new atypical active OT, and 5 eyes with recurrent atypical OT. The mean patient age was 41.2 ± 17.9 years (range, 15–78 years). Eighteen patients (47.4%) were women and 20 (52.6%) were men.

All OCT images included in the study met reading center criteria for sufficient image quality, meaning that the retinal layers could be analyzed and segmented with greater than 90% confidence.

### Overall Presence of SRF

In total, 20 eyes from 20 subjects demonstrated SRF in the posterior pole that was confirmed by SD-OCT, resulting in a presence of SRF as 51.3% (or 20/39) in active OT. Among them, 15 eyes with typical active OT, 1 with new atypical active OT and 4 eyes with recurrent atypical OT were detected. Thirteen eyes with SRF were found in the macula and 12 involving the fovea.

Thirteen eyes with SRF associated with retinal necrosis, 3 eyes with CME, and 3 eyes with CNV were observed. Characteristics related to SRF were shown in Table 1. The mean maximum height of SRF was 161.0 (standard deviation (SD): 130.4, range: 23–478) μm. The mean total volume of SRF was 0.47 (SD: 0.91, range: 0.005–4.12) mm^3^. A total of 40.0% eyes had SRF smaller than 0.03 mm^3^ in volume.

**Table 1:** General Characteristics of Patients with Active Ocular Toxoplasmosis.

Patient #	Age (Years)/ Gender	Eye	Subjective Complaints	Symptoms Duration	Snellen Visual Acuity	Lesion Category	Lesion Location /Zone	Lesion Location /Area	Related Feature	Mean Retina Thickness in Central subfield† Mean ∣SD (μm)		Total Retina Volume (mm^3^)	SRF Maximum Height (μm)	SRF Total Volume (mm^3^)
#1	21/woman	OD	Central see 'gray bar'	7d	20/200	typical active OT	zone 1	macula involving fovea	retinal necrosis	489.9	95.5	11.39	478	4.12
#2	48/woman	OD	VA drop	7d	20/40	typical active OT	zone 1	macula involving fovea	retinal necrosis	314.5	85	7.99	233	0.27
#3	30/man	OD			20/500	typical active OT	zone 1	macula involving fovea	retinal necrosis	227.4	44.7	8.19	38	0.02
#4	24/woman	OS	Black spots, flashes of light	7d	20/800	typical active OT	zone 1	macula involving fovea	retinal necrosis	320.1	44.4	7.55	64	0.02
#5	55/man	OS	Red eye, VA drop	1d	20/200	typical active OT	zone 1	macula involving fovea	retinal necrosis	448.4	46.4	10.04	55	0.03
#6	33/man	OS	VA drop	2m	NA	typical active OT	zone 1	macula involving fovea	retinal necrosis	677.9	73.8	9.23	163	0.03
#7	66/woman	OS			NA	typical active OT	zone 1	macula involving fovea	retinal necrosis	157.4	32.6	5.74	57	0.02
#8	52/woman	OS	VA drop	7d	1/35	typical active OT	zone 1	macular not involving fovea	retinal necrosis	113.1	26.9	6.40	78	0.06
#9	23/woman	OS	VA drop, eye pain, headache	4d	20/20	typical active OT	zone 2	superior to macula	retinal necrosis	240.3	20.6	7.62	180	0.84
#10	74/woman	OD	Pressure sensation, VA drop, headache	5d	20/40	typical active OT	zone 1	superior to ONH	retinal necrosis	270.5	26.5	7.97	70	0.2
#11	19/woman	OS	Central black spot	10d	20/20	typical active OT	zone 2	superior to macula	retinal necrosis	231.1	27.1	9.18	62	0.1
#12	18/man	OD		5d		typical active OT	zone 2	extramacula	retinal necrosis	257.5	32.2	8.82	410	1.29
#13	50/man	OD	VA drop		20/100	typical active OT	zone 1	macula involving fovea	retinal necrosis	238.8	36.8	7.30	33	0.02
#14	28/man	OD	Black spot	3d	20/100	typical active OT	zone 2	superior to macula	CME	887.7	86	13.66	138	0.02
#15	60/man	OS	Longstanding VA loss		20/400	typical active OT	zone 2	extramacula	CME	493.7	55.8	13.69	369	0.94
#16	55/man	OS	Longstanding VA loss		20/320	recurrent atypical active OT	zone 1	macula involving fovea	CME	410.2	55.7	10.59	280	0.2
#17	47/woman	OD			20/25	recurrent atypical active OT	zone 1	macula involving fovea	CNV	160.9	30.7	6.03	23	0.005
#18	70/man	OS	Sudden VA loss	4d	20/500	recurrent atypical active OT	zone 1	macula involving fovea	CNV	248.1	41.8	7.67	223	0.49
#19	39/woman	OD	Vision distortion, headache	10d	20/40	recurrent atypical active OT	zone 1	macula involving fovea	CNV	243.8	52.1	6.82	105	0.19
#20	30/man	OD	Eye pain	7d	20/20	new atypical active OT	zone 1	superior to ONH	neuritis	240.3	23.5	7.78	160	0.42

**†**Central subfield is defined as the subfield in the center of an ETDRS (early treatment diabetic retinopathy study) grid.

OT = ocular toxoplasmosis; OD = right eye; OS = left eye; VA = visual acuity; d = days; m = months; NA = not available; ONH = optic nerve head, CME = cystoid macular edema; CNV = choroidal neovascularization; SRF = subretinal fluid.

### Correlation of SRF with Retinal Necrosis

**Fig 3:**
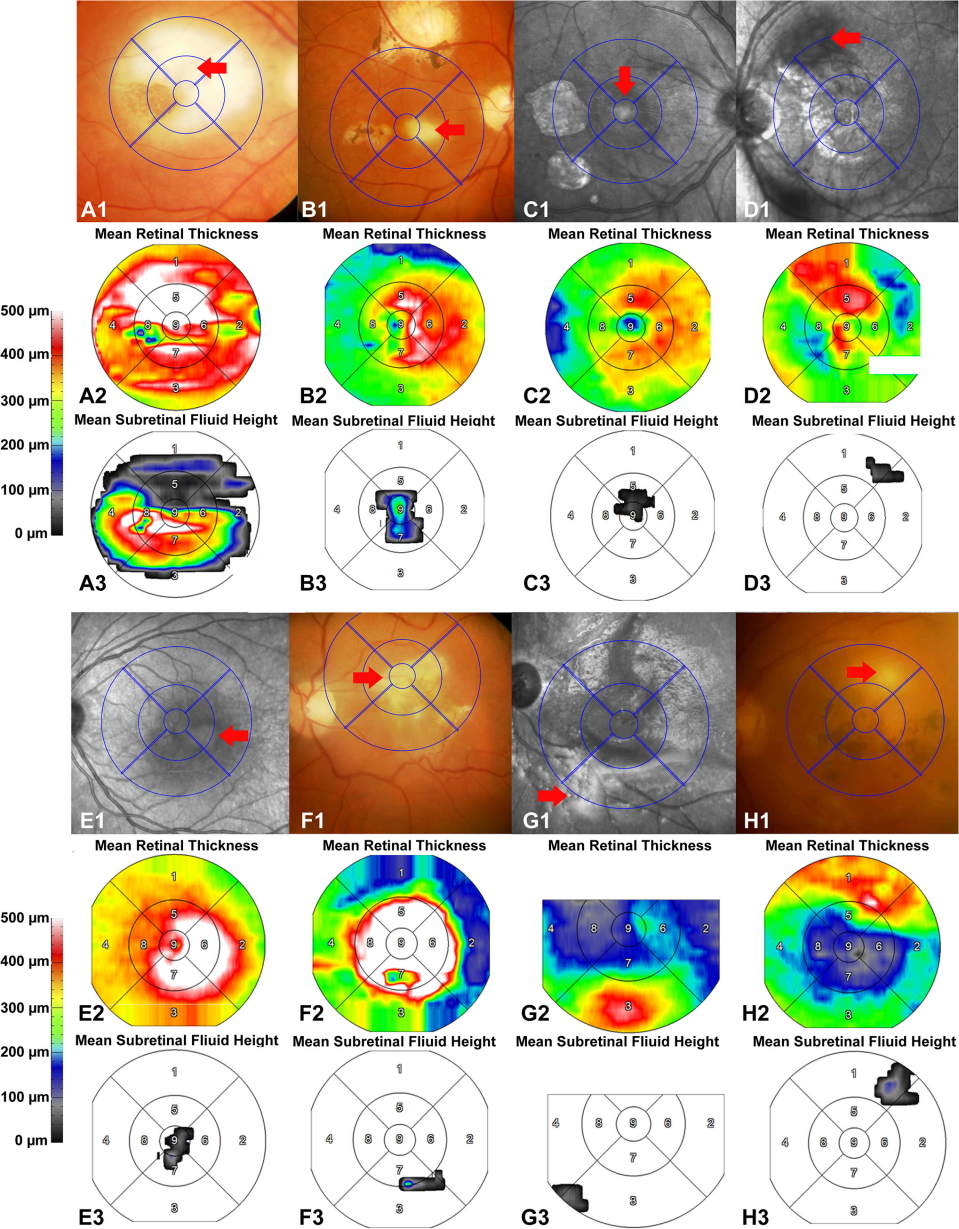
Subretinal fluid (SRF) in eyes with typical active ocular toxoplasmosis associated with retinal necrosis in the macula as seen on optical coherence tomography (OCT) (Patients 1–8, Table 1). **Parts A1-H1**: fundus photos (FP) or infrared (IR) images of the macula overlying with early treatment diabetic retinopathy study (ETDRS) grid, for eyes with active OT. Red arrows in each eye indicated areas with active retinitis. **Parts A2-H2:** mean retinal thickness maps shown in the ETDRS grid corresponding to the overlapped grid in Figure A1-H1. **Parts A3-H3:** maps of mean SRF height shown in ETDRS grid corresponding to the overlapped grid in Figure A1-H1. 1 = superior outer macula; 2 = temporal outer macula; 3 = inferior outer macula; 4 = nasal outer macula; 5 = superior inner macula; 6 = temporal inner macula; 7 = inferior inner macula; 8 = nasal inner macula; 9 = central subfield.

Thirteen eyes from 13 patients with SRF demonstrated evidence of retinal necrosis (Patients 1–13, Table 1, Fig 3), resulting in a prevalence of 33.3% (or 13/39) in all included eyes, or 39.4% (or 13/33) in eyes with typical active OT, or 65.0% (or 13/20) in eyes with SRF found in the study. All these eyes had lesions consistent with typical active OT, of which, 9 were located in the macula and 4 outside the macula but within posterior pole. All pockets of SRF were adjacent to the active necrotic lesions, except for one eye (Patient 13). At baseline, the mean maximum height of SRF was 147.8 (SD: 139.7, range: 33–478) μm with a mean total volume of SRF as 0.54 (SD: 1.10, range: 0.02–4.12) mm^3^. One patient was transferred to the local ophthalmologist without further follow-up in our clinic. For the remaining 12 eyes, SRF was observed with complete absorption after conventional anti-toxoplasmosis treatment during the study period. The mean duration for observation of SRF clearance was 33.8 (SD: 22.2, range: 7–84) days. The mean rate of SRF clearance was 0.0128 (SD: 0.0198, range: 0.0002–0.0665) mm^3^/day.

### Representative Case—Patient 9

A 23 year-old Caucasian woman, with a previous diagnosis of OT in the left eye, presented with new-onset “foggy vision” and pain in the left eye, in addition to persistent headache for a four-day period (Patient 9, Table 1, Fig 1). On presentation, VA was 20/20 in the left eye with 1+ vitreous cell. Dilated fundus examination revealed a normal macula and optic nerve head (ONH), but with evidence of whitish-yellow inflammatory lesion superior to the macula, in association with intraretinal hemorrhage and surrounding SRF. On OCT scanning though the fovea, vitreous cells were present without any retinal abnormality. On OCT scans through the active lesion, however, full thickness retinal interruption, which is corresponding to the retinal necrosis seen by fundus examination and greatly increased choroidal thickness were observed. SRF presented adjacent to the necrotic lesion, with a maximum height of 180 μm and a total volume of 0.84 mm^3^. The patient was commenced on oral clindamycin (300 mg 4 times daily) immediately and Prednisolone (75mg/d tapered) starting two days later.

After six weeks, the patient returned with a vision of 20/20 in the left eye. On fundus examination, the prior active lesion was noted to be smaller in size and without visible SRF. OCT scanning through the lesion showed no evidence of SRF, reduced numbers of vitreous cells, and decreased choroidal thickness compared to initial presentation.

### Correlation of SRF with CME

A total of 3 eyes from 3 patients (Patients 14–16, Table 1) showed SRF in association with CME. Presence of SRF and CME (also with huge intraretinal cystoid lesion) superior to the macula adjacent to a typical active lesion was observed in Patient 14. Coexistence of SRF and CME in the macula, away from a typical active lesion in the peripheral retina was documented in Patient 15. SRF accompanied by CME in the macula in one eye with a previous diagnosis of OT without sign of uveitis activity in the current visit was seen in Patient 16. One eye (Patient 14) responded well to oral antibiotic treatment alone with complete absorption of SRF and CME (including the huge intraretinal cystoid lesion) seen after 25 days of therapy. However, two other eyes presented with long-standing CME with SRF lasting for months or years, despite conventional treatment and combined intravitreal injections (Patient 15 and16).

### Representative Case—Patient 16

A 55 year-old Caucasian man presented on January 21 2011, with complaints of 'persistent decreased vision' in the left eye for a two-year period (Patient 16, Table 1, Fig 2). The patient was initially diagnosed with panuveitis secondary to OT in 1987 and had recurrences a number of times subsequently. The eye was complicated by cataract, epiretinal membrane (ERM) and CME, and has been treated with pars plana vitrectomy (PPV) with ERM peeling on November 29 2010 in a separate center. Forty-five days following vitrectomy, she also underwent cataract removal (phacoemulsification), intraocular lens implantation and release of posterior iris synechiae. However, she had no vision improvement afterwards.

On presentation to our clinic, VA was 20/320 in the left eye. Anterior examination of the left eye showed slight conjunctival hyperemia, a clear cornea with endothelial precipitates, a deep anterior chamber with Tyndall+, a round and responsive pupil, and the intraocular lens in proper position. Fundus examination revealed macular edema and a single old retinochoroidal scar in the superior nasal peripheral retina. SD-OCT demonstrated increased retinal thickness with CME and SRF(Table 1). On March 16, she was given oral acetazolamide 125 mg three times daily for 14 days. However, the CME and SRF remained unchanged 3 and 6 months after the initial visit. She was then given intravitreal injections of 1.25 mg Bevacizumab on 07.13.2011, 12.14.2011, and 03.06.2012, respectively. On 06.11.2012, she presented with a VA of 20/400 and complete absorption of SRF, however, with persistence of CME. She was then given an intravitreal injection of Dexamethasone implant 0.7mg on the same day. One month later, the CME had resolved on OCT, although without subjective vision improvement. However, CME recurred two months subsequently and she was given additional intravitreal injections of Dexamethasone implant on 09.10.2012, 01.28.2013, and 02.25.2013, respectively. During this time, CME was somewhat improved on one occasion (02.25.2013) but mainly remained unchanged till the end of the study period.

### Correlation of SRF with CNV

Three eyes from 3 patients with SRF in association with CNV were found in our study (Patient 17–19, Table 1). SRF volume in Patient 17 was 0.005 mm^3^ at baseline and persisted in size till the last available visit (26 months after baseline). SRF in Patient 18 had a total volume of 0.49 mm^3^ at baseline and slowly decreased in amount until it completely resolved (31 months after baseline). SRF in Patient 19 presented with a total SRF volume of 0.19 mm^3^ at baseline and was resolved 8 months later. The patient had no further follow-up in our clinic.

### SRF related to Other Manifestations

One eye presented with SRF unrelated to necrosis, CNV or CME (Patient 20, Table 1). The patient presented with 'neuritis' and was confirmed by laboratory testing (serology and aqueous humor analysis showed detection of intraocular Toxoplasma gondii antibodies with Goldmann/Witmer coefficient >3) to be atypical new active toxoplasmosis. SRF was detected superior to ONH, i.e. adjacent to the active lesion.

## Discussion

In this study, the presence and clinical course of SRF in eyes with active OT were evaluated using SD-OCT. Out of 39 eyes (38 patients) included in the study, 20 eyes from 20 subjects demonstrated SRF in the posterior pole that was confirmed by SD-OCT. SRF was evident in 45.5% (or 15/33) of eyes with typical active OT and in 51.3% (or 20/39) of active OT in the current study. In total, 65.0% (or 13/20) of the cases with SRF were associated with retinal necrosis, 15.0% (or 3/20) with CME and 15.0% (or 3/20) with CNV.

Ocular toxoplasmosis classically appears as retinochoroidtitis, which is a focus of retinitis arising from the border of a retinochoroidal scar [21]_._ However, the phenotype can show considerable variation in atypical cases [21]_._ Knowledge of the various presentations of OT is important for the clinician and attention to the characteristics of OT may yield insights into disease mechanisms. In our study, "typical" OT was recognized and categorized based on the presence of active lesions of retinitis. One eye presented as neuritis without previous history of OT was defined as "new atypical active OT". Eyes with previous diagnosis of OT, which developed new symptoms without signs of active retinitis were referred as "recurrent atypical active OT" (e.g. CNV [22–26] and CME [1,12,21,22,27–30]), although a direct causative link cannot be made for sure.

SRF is seen by OCT as a hyporeflective space (i.e. detachment) between the neurosensory retina and the underlying RPE. Depending on the mechanism of SRF accumulation, RD has been classified into rhegmatogenous, tractional, and serous/exudative. Our study was intended to only describe cases with SRF associated with serous/exudative RD. The frequency of RD in OT was reported to be 5% or 6% by Friedmann, et al. and Bosch-Driessen, et.al, respectively [31, 8]. However, both reports only described tractional or rhegmatogenous RD in the study population, which made our study difficult to compare with their results. Some studies have mentioned SRF as a possible presentation of OT [5,6,9,10,31]. Until now, it has generally been thought that SRF is an uncommon feature of OT, and of many causes of serous detachment, OT is considered one of the least common [32]_._

Recent clinical observation and laboratory findings have led to a reassessment of many preconceived notions regarding OT.^14^ With the widespread availability of SD-OCT, SRF is now more frequently detected compared to the method with clinical observation alone or with stratus OCT [10]_._ Using SD-OCT, Diniz and colleagues observed SRF in 20% out of 10 eyes with active OT, which were overlooked by both Stratus OCT and clinical examination [10]_._ Foster et al. also reported the presence of SRF in 20.8% out of 24 eyes with active OT [33]_._ However, in these studies, the OCT scanning protocols covered a smaller area of the posterior pole. It is known that SRF in many lesions, especially those with small, is easily neglected if the OCT scanning is not performed close to the affected area. In our study, we could show that lesions with very little SRF (for example, total volume ≤ 0.3 mm^3^) were seen in the majority of cases. Thus, application of the OCT scanning through all involved areas in the posterior pole could possibly enhance the opportunity to detect SRF in our population. SRF was found in 45.5% of eyes with typical new active OT in our study, a presence much higher than expected based on the previous concept. Our results overall support the previous reports by Diniz et al. and Foster et al. that SRF is more commonly seen in active OT using SD-OCT. Yet, since many active OT lesions are in the peripheral retina, which is out of the reach of existing commercial OCT devices, our study using currently available SD-OCT systems still could not identify the true overall frequency of SRF or serous RD in OT. However, due to our extramacular OCT scanning protocol, the prevalence of SRF or serous RD in the posterior pole (or zone 1 plus zone 2) could be obtained reliably, at least in our cohort.

In eyes with typical (15 eyes) and new atypical (1 eye) active OT in our study population, the location of SRF is highly related to the site of active retinitis; only 2 eyes were noted to have SRF remote from the active lesion. This implies that the fluid accumulation in most cases was likely due to increased vascular permeability from the active inflammatory process of retinitis or retinal necrosis.

SRF related to retinal necrosis responded well to the anti-Toxoplasmosis treatment in our study: resolution of fluid was typically rapid after a short course of oral antibiotics with or without oral steroids. This process is independent of fluid volume, as resolution was observed even when the total volume of the SRF was relatively large (for example Patient 1). Although, for most of our patients, SRF was not the determining factor for vision recovery due to the central location of retinal necrosis; one would expect that when the necrosis doesn't involve the fovea, a vision improvement due to SRF should be seen after systemic anti-Toxoplasmosis therapy. On the other hand, SRF associated with CME demonstrated a different response to treatment. One eye with SRF and CME (including a large cystoid lesion) did respond favorably to systemic anti-toxoplasmosis therapy; however, two other eyes with long-standing SRF and CME did not improve despite combined convention and intravitreal treatment. Similarly for SRF related to CNV, the clearance of SRF appeared slow or incomplete. Although SRF in Patient 18 and 19 eventually resolved, this process took 936 days and 233 days, respectively. It is also not known whether Patient 19 had recurrence of CNV later on due to the loss of follow-up. For Patient 17, although the total volume of SRF was only 0.005 mm^3^ at baseline, it remained unchanged throughout the study period (2 years after the baseline).

Our study has many limitations that one would expect with a retrospective analysis. Although all OCT images were acquired according to a standard scanning protocol in our imaging unit, longitudinal assessment of fluid would best be accomplished with a prospective design with assessments and pre-specified standard intervals. Thus, our data must be interpreted with caution. Also, as discussed above, only a restricted area in the macula or posterior pole could be scanned thoroughly by our existing OCT system. Since many lesions with SRF could be missed without SD-OCT examination and OT may present with additional peripheral lesions, some areas with activity were possibly overlooked even though extramacular scanning protocol was utilized in our study.

## Conclusion

In this study, we have observed that SRF or serous RD was much more commonly seen by SD-OCT in active OT, with a prevalence as 45.5% (or 15/33) in eyes with typical active OT or 51.3% (or 20/39) in eyes with all active OT. In eyes with typical and new atypical active OT, the location of SRF is highly related to the site of active retinitis, which implies that the fluid accumulation in most cases was likely due to increased vascular permeability from the active inflammatory process of retinitis or retinal necrosis. The majority of cases with SRF was associated with retinal necrosis and reacted well to the anti-toxoplasmosis therapy, regardless of baseline total fluid volume. However, SRF accompanying with CME or CNV responded less favorably or remained refractory to conventional or combined intravitreal treatment, even when the amount of SRF was small. Further studies are needed to verify its relevance.
