# Correction: Subretinal Fluid in Eyes with Active Ocular Toxoplasmosis Observed Using Spectral Domain Optical Coherence Tomography

**DOI:** 10.1371/journal.pone.0134074

**Published:** 2015-07-21

**Authors:** Yanling Ouyang, Fuqiang Li, Qing Shao, Florian M. Heussen, Pearse A. Keane, Nicole Stübiger, Srinivas R. Sadda, Uwe Pleyer

There is an error in the “Participants” subsection of the Abstract. The correct sentence is: Thirty-nine eyes from 38 patients with active OT.
